# A Feedback Model of Attention Explains the Diverse Effects of Attention on Neural Firing Rates and Receptive Field Structure

**DOI:** 10.1371/journal.pcbi.1004770

**Published:** 2016-02-18

**Authors:** Thomas Miconi, Rufin VanRullen

**Affiliations:** 1 Centre de Recherche Cerveau et Cognition (CerCo), CNRS & Université Paul Sabatier, Pavillon Baudot CHU Purpan, Toulouse Cedex, France; 2 The Neurosciences Institute, La Jolla, California, United States of America; University of Tuebingen, GERMANY

## Abstract

Visual attention has many effects on neural responses, producing complex changes in firing rates, as well as modifying the structure and size of receptive fields, both in topological and feature space. Several existing models of attention suggest that these effects arise from selective modulation of neural inputs. However, anatomical and physiological observations suggest that attentional modulation targets higher levels of the visual system (such as V4 or MT) rather than input areas (such as V1). Here we propose a simple mechanism that explains how a top-down attentional modulation, falling on higher visual areas, can produce the observed effects of attention on neural responses. Our model requires only the existence of modulatory feedback connections between areas, and short-range lateral inhibition within each area. Feedback connections redistribute the top-down modulation to lower areas, which in turn alters the inputs of other higher-area cells, including those that did not receive the initial modulation. This produces firing rate modulations and receptive field shifts. Simultaneously, short-range lateral inhibition between neighboring cells produce competitive effects that are automatically scaled to receptive field size in any given area. Our model reproduces the observed attentional effects on response rates (response gain, input gain, biased competition automatically scaled to receptive field size) and receptive field structure (shifts and resizing of receptive fields both spatially and in complex feature space), without modifying model parameters. Our model also makes the novel prediction that attentional effects on response curves should shift from response gain to contrast gain as the spatial focus of attention drifts away from the studied cell.

## Author Summary

Exerting visual attention results in profound changes in the activity of neurons in visual areas of the brain. Attention increases the firing of some neurons, decreases that of others, moves and resizes the receptive fields of individual neurons, and changes their preferred features according to what is being attended. How are these complex, subtle effects generated? While several models explain various subsets of these effects, a consistent explanation compatible with anatomical and physiological observations remains elusive. Here we show that the apparently complex and multifaceted effects of attention on neural responses can be explained as the automatic consequence of a top-down modulation, falling on higher visual areas (as suggested by anatomical observations), and interacting with short-range inhibition and feedback connections between areas. Our model only assumes the existence of well-known features of brain organization (reciprocal inter-area connections, mutual inhibition between neighboring neurons) to explain a wide range of attentional effects, including apparently finely-tuned effects (complex shifts in feature preferences, automatic scaling of competitive effects to receptive field size, resizing or shifting of receptive fields, etc). Our model also makes novel, testable predictions about the effect of certain attentional manipulations on neural responses.

## Introduction

Attention modulates the responses of visual neurons in diverse ways [1–4]. Some studies suggest that spatial attention produces a response-gain effect on neural responses (overall multiplication of the response curve as a function of contrast, with maximal effect at highest contrast), while others suggest a contrast gain (leftward shift of the response curve, with little effect at highest contrasts) [5,6]; yet other studies report ambiguous results [7]. Attention also biases the competition between different stimuli occurring within the receptive field (RF) of a given cell, in such a way that the actual response is more similar to that elicited by the selected stimulus in isolation [8]. Interestingly, the spatial range of these competitive effects seems restricted to the size of the RF, across several visual areas with very different typical RF sizes [9–11] (importantly, note that this within-RF suppression is distinct from outside-RF, “surround” suppression, not just positionally, but also in tuning, strength and presumably mechanism; see Discussion).

Attention can also be directed towards a specific feature dimension, rather than a specific position. Such feature-based attention increases the response of neurons selective to the attended feature, while reducing the responses of neurons selective to non-attended features—the so-called “feature-similarity gain principle” [12].

In addition to these effects on firing rates, attention also modifies the structure and size of receptive fields, both in topological and featural space. Spatial attention shifts RF position towards the focus of attention [13,14]. Intriguingly, focusing attention within the RF tends to shrink the RF (in addition to shifting its center), while attending to the same stimulus located just outside the RF tends to expand the RF towards the focus of attention [15]. Similarly, feature-based attention can also shift receptive fields in feature space: the featural receptive field of individual neurons is subtly altered to better match the features of the attended stimulus [16,17]. Note that this is quite different from the previously mentioned sharpening of tuning curves due to a feature-similarity gain effect: feature-similarity gain makes certain neurons fire more (or less) for all stimuli, while the subtle shift of RFs in feature space makes all neurons respond more to certain stimuli and less to others, in such a way that neurons seem to be more tuned to the complex attended stimulus.

Several authors have suggested that these attentional effects are compatible with a multiplicative modulation being selectively applied to specific *inputs* of the recorded neurons, interacting with appropriately scaled divisive lateral inhibition [4,18–23]. A particularly detailed example is Reynolds and Heeger’s so-called “normalization model” of attention [4], which shows both contrast-gain and response-gain effects, as well as biased competition and feature-similarity gain. While Reynolds and Heeger do not mention RF alterations, other authors have shown that the same general principle can explain RF shifts [19,21].

However, experimental studies consistently report that attentional effects occur earlier and more strongly in higher (downstream) areas, and later and more weakly in lower (input) areas of visual cortex—a so-called “backwards progression of attentional effects” [24]. Attentional modulation of single-cell responses [9,24,25] and human imaging responses [26] increases in magnitude at higher levels of the visual hierarchy, from V1 to V4 (though attentional effects are still measurable and significant in V1 [27,28]). Meanwhile, the onset of attentional modulation on single-cell responses occurs progressively later in V4, V2, then V1 [24].

Furthermore, areas that are thought to control attention (especially Frontal Eye Fields—FEF, and Lateral Intraparietal cortex—LIP) [2,29] seem to target higher areas of the visual system, rather than input areas such as V1. FEF has strong, “feedforward-like” projection towards V4, but not V1 [30]; FEF synchronizes with V4 during attentional tasks, with FEF leading V4 [31], and microstimulation of FEF produces effects in V4 (spatially localized increases and decreases in responses) that are congruent with those of attention [32]. Similarly, LIP is strongly connected to V4 and MT, but not V1. LIP synchronizes with the Middle Temporal (MT) area of dorsal visual cortex during attention, with LIP leading [33]. LIP-to-dorsal visual cortex modulation was also observed in human fMRI data [34].

In addition, the input-multiplication hypothesis does not explain why competitive effects are scaled to RF size (this is simply assumed in the normalization model) or why RF scaling should shift from shrinking to expansion as attentional focus moves outside the RF. Furthermore, it is not specified how attentional modulation would know which inputs must be modulated to produce the correct RF shift in the studied cell—a very simple problem for spatial attention, but a more daunting one for the subtle featural effects observed in complex-selectivity V4 cells [17].

Some models implement attention as a top-down modulation of higher-level areas, as suggested by anatomy and physiology [35,36]. As expected, in such models, attentional effects are stronger and earlier in higher areas, and smaller and later in lower areas. However, top-down modulation leads to the erroneous prediction that RFs should shift *away* from the focus of attention, both in topological and feature space [37]; this is because the modulated cells would inhibit their neighbours, with greater inhibition when the stimulus comes closer to the focus of attention (and thus excites more the modulated cells). While RF shifts can be produced by invoking appropriately-tuned lateral connections and attentional surround inhibition [37], this leads to a prediction of RF *expansion* rather than shrink when attention falls directly on the RF. It is also not clear how lateral connections can explain the very long range of RF attraction effects, which extend beyond V4 RFs [15].

**Fig 1:**
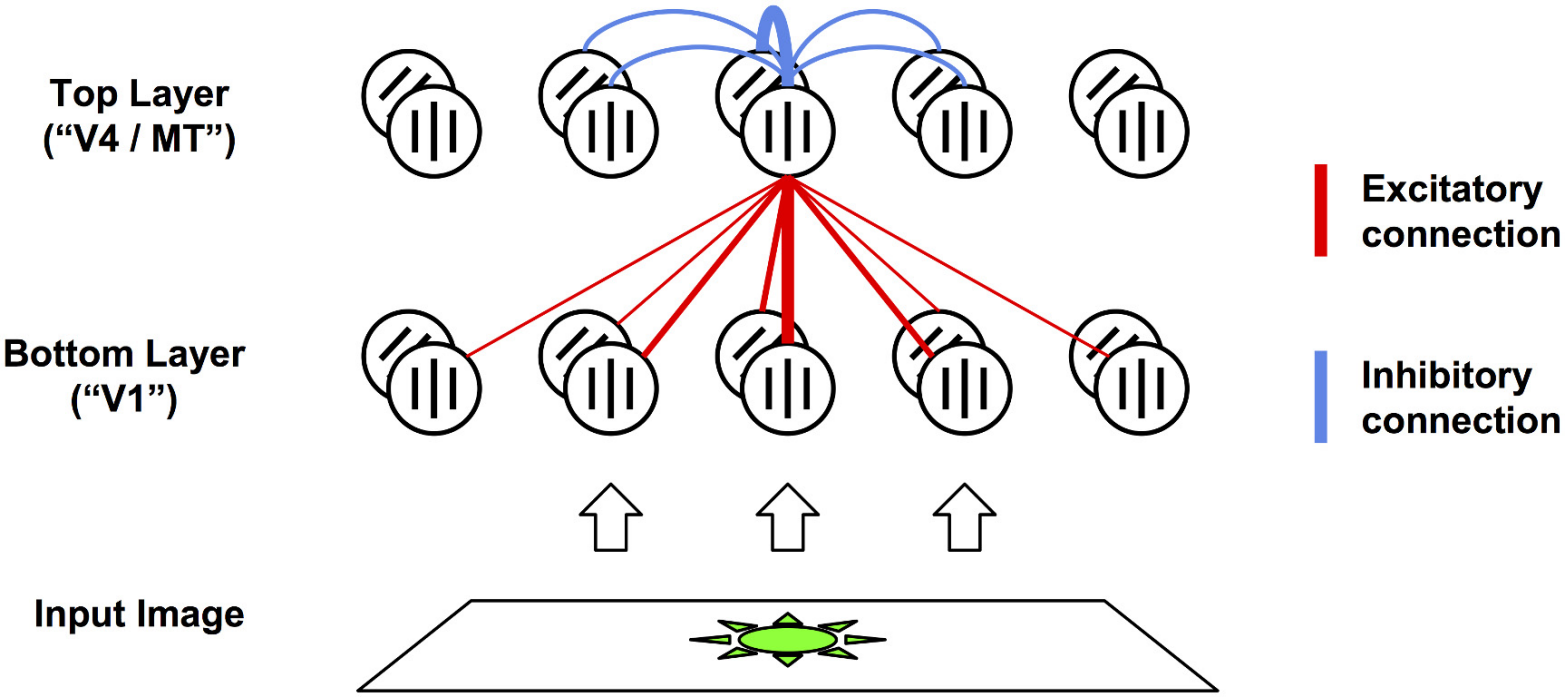
Model sketch. Each layer is composed of orientation selective cells (8 per location; only 2 shown for clarity). The bottom layer processes image inputs. The top layer receives excitatory connections from the bottom layer, inversely proportional to distance and difference in preferred orientation (red lines). The top layer sends feedback connections to the bottom layer, with weights proportional to those of feedforward connections, but modulatory (multiplicative) rather than driving (additive). In addition, all cells receive divisive short-range inhibition from their neighbour, decaying quickly with distance (blue lines). For clarity, we only show connections to and from one single top-layer cell; other cells have similar connectivity.

**Fig 2:**
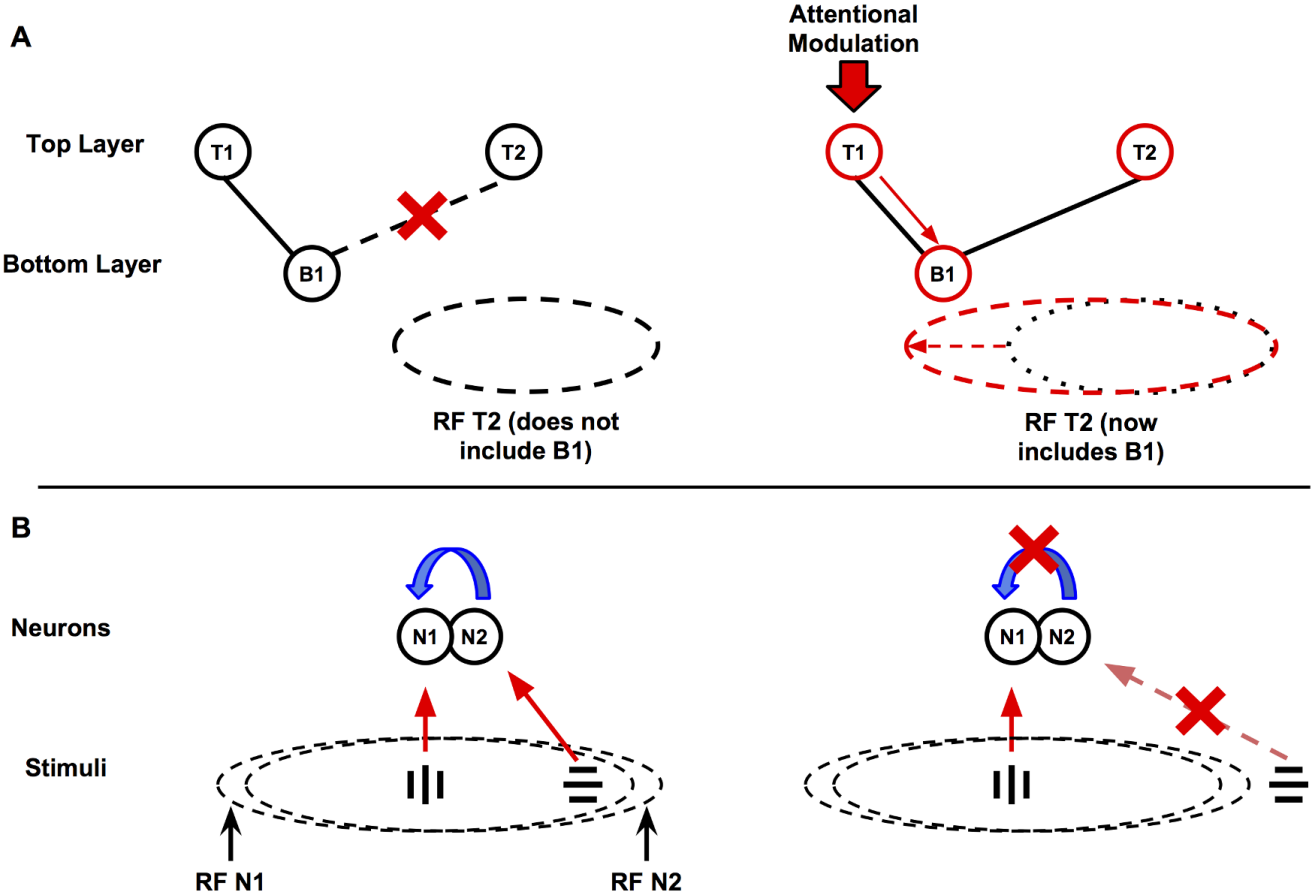
Explanation of model mechanisms. Attentional modulation on top-layer cells extends the RF of other top-layer cells, and short-range inhibition automatically scales competition to RF size. Top panel (A): Attention expands RFs. Without attention, cell B1 is too far from T2 to excite it significantly, and thus falls outside T2’s RF (Left). In the presence of attention (Right), the top-down modulation is propagated through feedback connections to B1, making it respond more strongly to a given stimulus. As a result, B1 can now reliably excite T2, and thus is now part of its RF. Bottom panel (B): Short-range inhibition scales competition to RF size. A non-preferred stimulus in N1’s RF excites cells close to N1, which in turn inhibit N1 through short-range inhibition (Left). But if the same stimulus falls well outside N1’s RF, it will also fall outside the RF of cells close to N1, which are the only ones that can inhibit N1; therefore, N1 will not be inhibited by the non-preferred stimulus (Right). The only required assumptions are that neighbouring cells tend to have comparable RF sizes, and that the spatial extent of short-range lateral inhibition is small relative to RF size.

Here we propose a simple mechanistic model of attentional modulation, which explains how a top-down modulatory signal, falling upon the upper areas of the visual hierarchy, can produce all the previously mentioned effects of attention on neural responses (Fig 1). Our model only requires the existence of modulatory feedback connections between areas, and short-range mutual inhibition within each area. Briefly, we suggest that the observed effects of attention arise spontaneously from the effect of feedback connections between higher and lower areas, interacting with short-range divisive mutual inhibition between neighbouring cells within each area. Feedback connections (with weight proportional to the feedforward connection between any two cells, but modulatory rather than driving) redistribute the top-down modulation to lower areas; this in turn alters the inputs of other higher-area cells, including those that did not receive the initial modulation. This produces the effects of attention on neural responses, including receptive field expansion towards the focus of attention (Fig 2A). Simultaneously, short-range lateral inhibition between neighbouring cells produce competitive effects that are automatically scaled to RF size in any given area; because under short-range lateral inhibition, a stimulus can only inhibit a given cell A if it activates a nearby cell B, which (because it is nearby) is very likely to have an RF highly similar in position and size to that of A (Fig 2B).

Our model explains all effects of visual attention described above, on response rates and RF structure, both in topological and fine feature space. In addition, the model makes a novel prediction: attentional effects on response curves should turn from response gain to contrast gain as the focus of attention shifts away from the center of the studied cell’s RF.

## Results

### Attentional effects on firing rates

**Fig 3:**
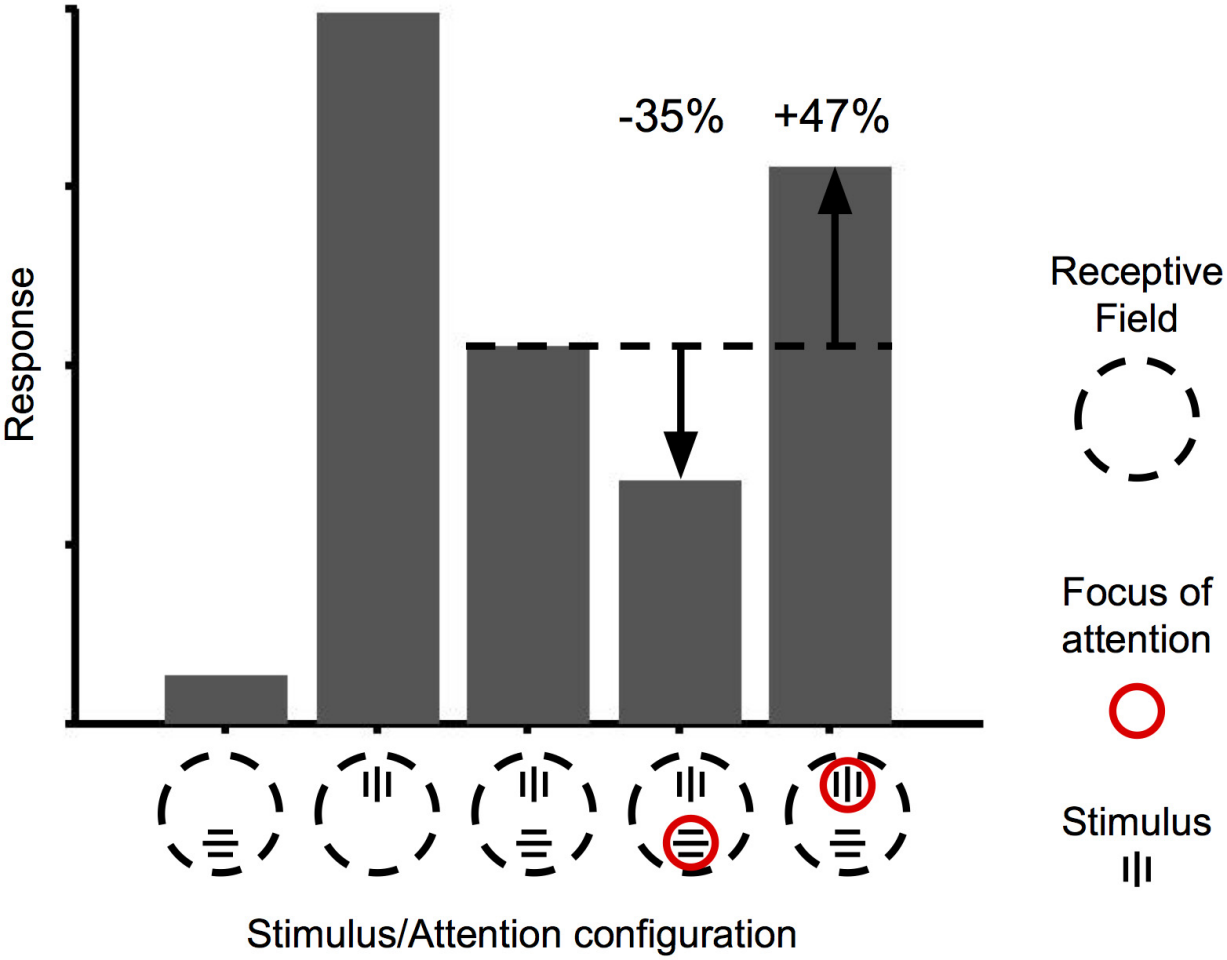
Biased competition. Response of a top-layer cell selective for vertically-oriented stimuli. Response to two opposite stimuli in the RF (third bar) is intermediate between the cell’s responses to either stimulus in isolation (first and second bar). Attending to one of the stimuli partially restores the response to that stimulus alone, without any change in the display (fourth and fifth bar). The numbers above the fourth and fifth bars indicate the magnitude of attentional modulation in comparison to the no-attention response to the exact same stimulus (third bar).

We first investigated the response of a top-layer cell to two stimuli (one preferred and one anti-preferred) falling within the receptive field—that is, a “biased competition” experiment [8]. The stimulus consists of two semi-rectified Gabor functions, one vertically oriented and one horizontally oriented, on opposite sides and at equal distances from the center of the studied cell’s RF. Fig 3 shows the response of the studied cell to either stimulus in isolation, to both stimuli together in the absence of attention, and to both stimuli with spatial attention focusing on one or the other stimulus. As expected, response to both stimuli was intermediate between the responses to either stimulus in isolation, and attention partially restored response to the attended stimulus—either increasing or reducing the cell’s response to an unchanged visual input. The amount of attentional modulation in either direction was approximately +/- 30%, compatible with experimental observations [9,20].

As expected, attentional modulation was stronger in the top layer than in the bottom layer: maximum attentional modulation (right under the attentional focus) was 51% for the top layer, versus 7% in the bottom layer. Note that the latter figure confirms the weak effect of attention on V1 firing rates.

**Fig 4:**
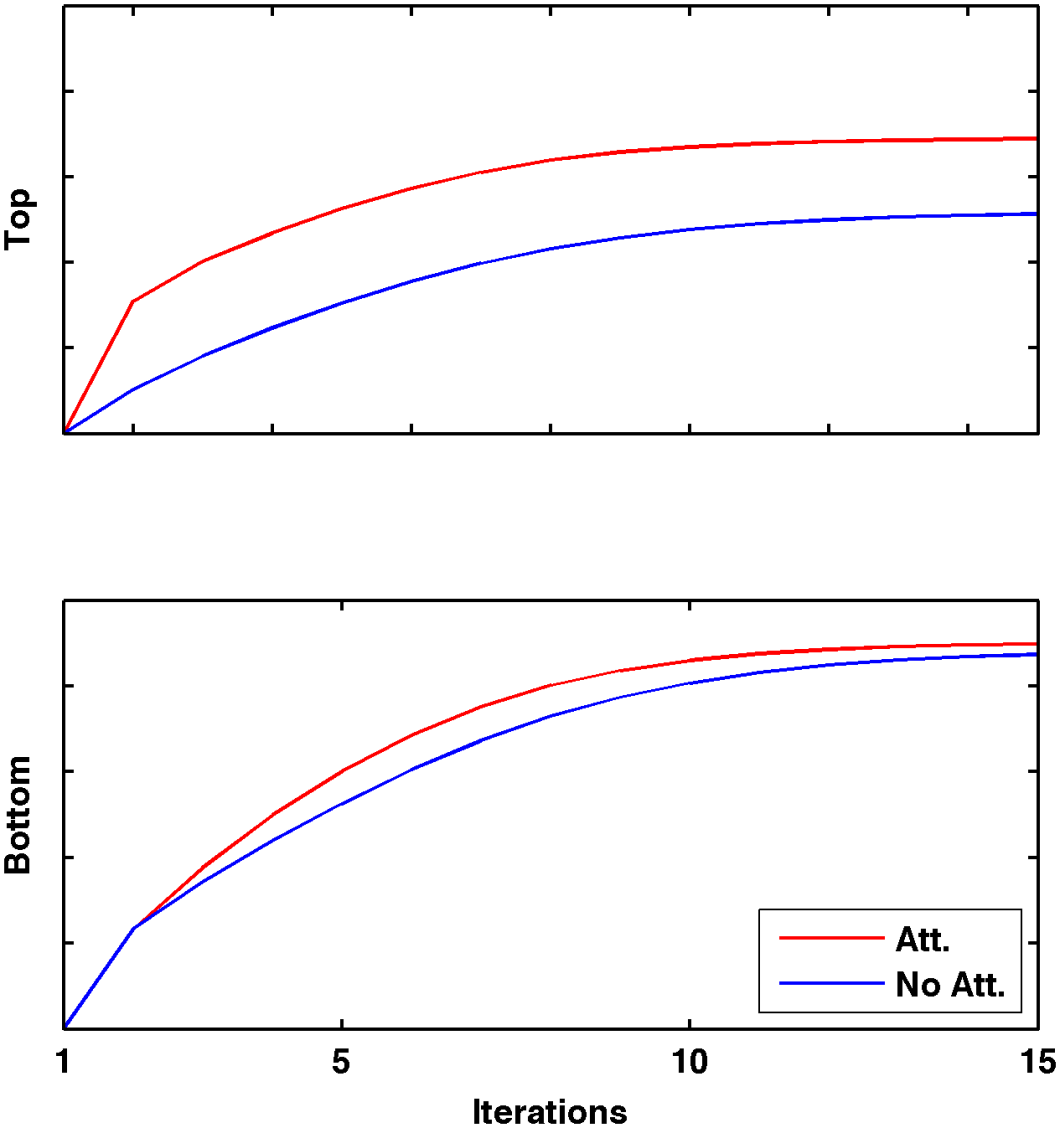
Time course of attentional effects. Cell activations as a function of time in the top and bottom layer, in the presence or absence of attention. Attentional modulation occurs earlier and is stronger in the top layer than in the bottom layer.

To better characterize the different dynamics of attentional modulation in either layer, we ran a simple experiment with a single semi-rectified Gabor function, measuring the activation of bottom-layer and top-layer cell selective to the stimulus’ orientation, both with and without focusing spatial attention on the stimulus (Fig 4). Our model computes cell activations iteratively, computing first the bottom-layer activations, then the top-layer activations, then iterating after applying feedback from the top to the bottom layer, until activations equilibrate (see Materials and Methods). As expected, attentional modulation occurs first in the top layer. Furthermore, attentional effects are much stronger in the top layer than in the bottom layer, in accordance with experimental results [9,24]. Note that, since neural activations are computed instantaneously at each pass, we can only document gross dynamics at the scale of entire layers; our model cannot capture more fine-grained dynamics at single-cell level, such as noise correlations (see Discussion).

**Fig 5:**
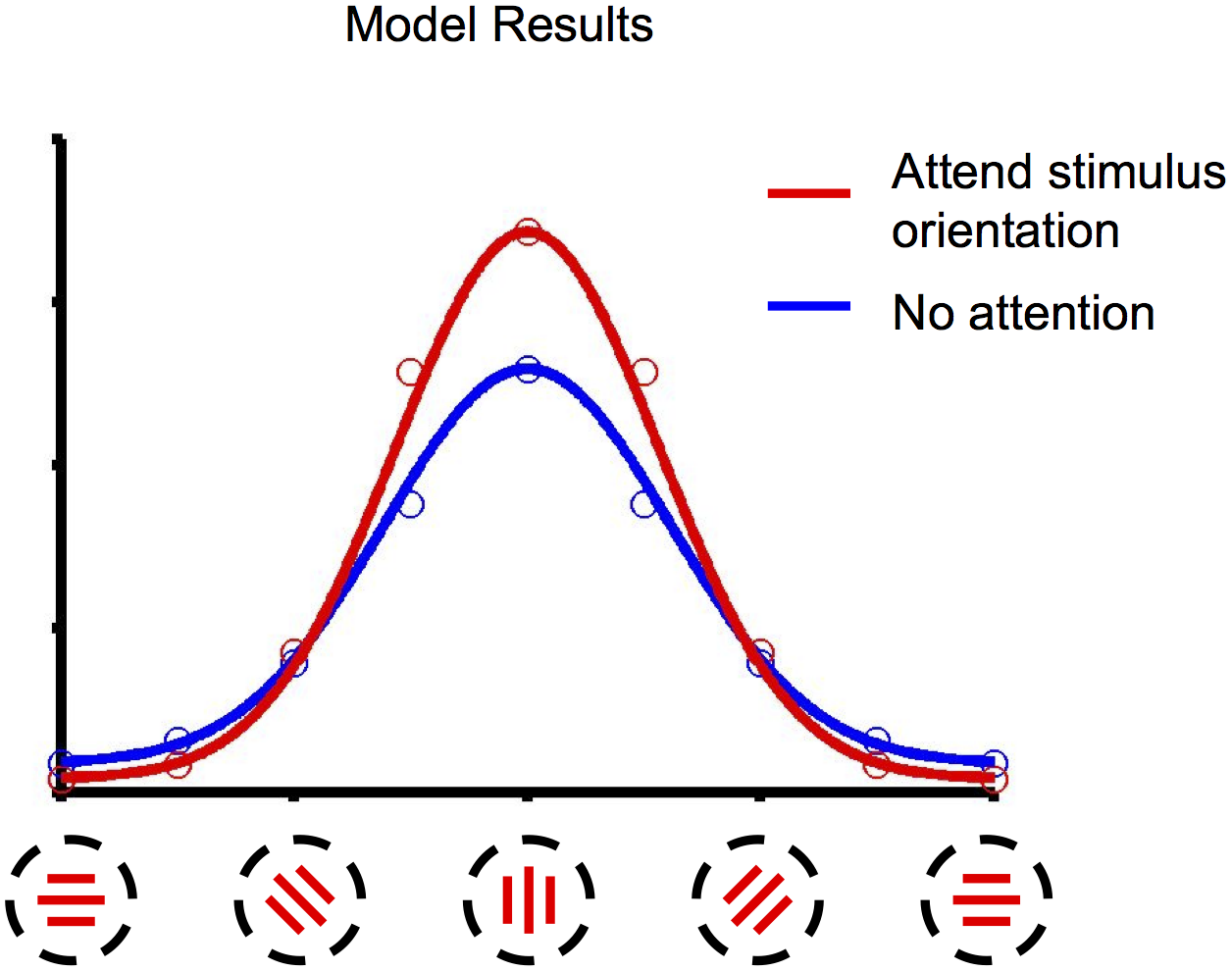
Feature-similarity gain. Response of a model cell selective for vertically-oriented stimuli, for stimuli of varying orientation, with feature-based attention to the stimulus orientation (red) or in the absence of attention (blue). Dots represent model responses; lines are Gaussian fits. Compare Fig 4 of [12].

We then studied the responses of a top-layer cell in a feature-based attention scenario. The visual input was a single semi-rectified Gabor function, centered at the center of the cell’s RF, taking successively 8 different orientations from 0 to 157.5 degrees inclusive. Attention was always allocated to the stimulus orientation. This emulates the experiment performed by Martinez-Trujillo and Treue [12]. Our model reproduces the observed result of a sharpened tuning curve, in which attention enhances response when the stimulus is preferred and reduces it when the stimulus is anti-preferred (see Fig 5). Thus, our model reflects the similarity-gain principle posited by Martinez-Trujillo and Treue.

**Fig 6:**
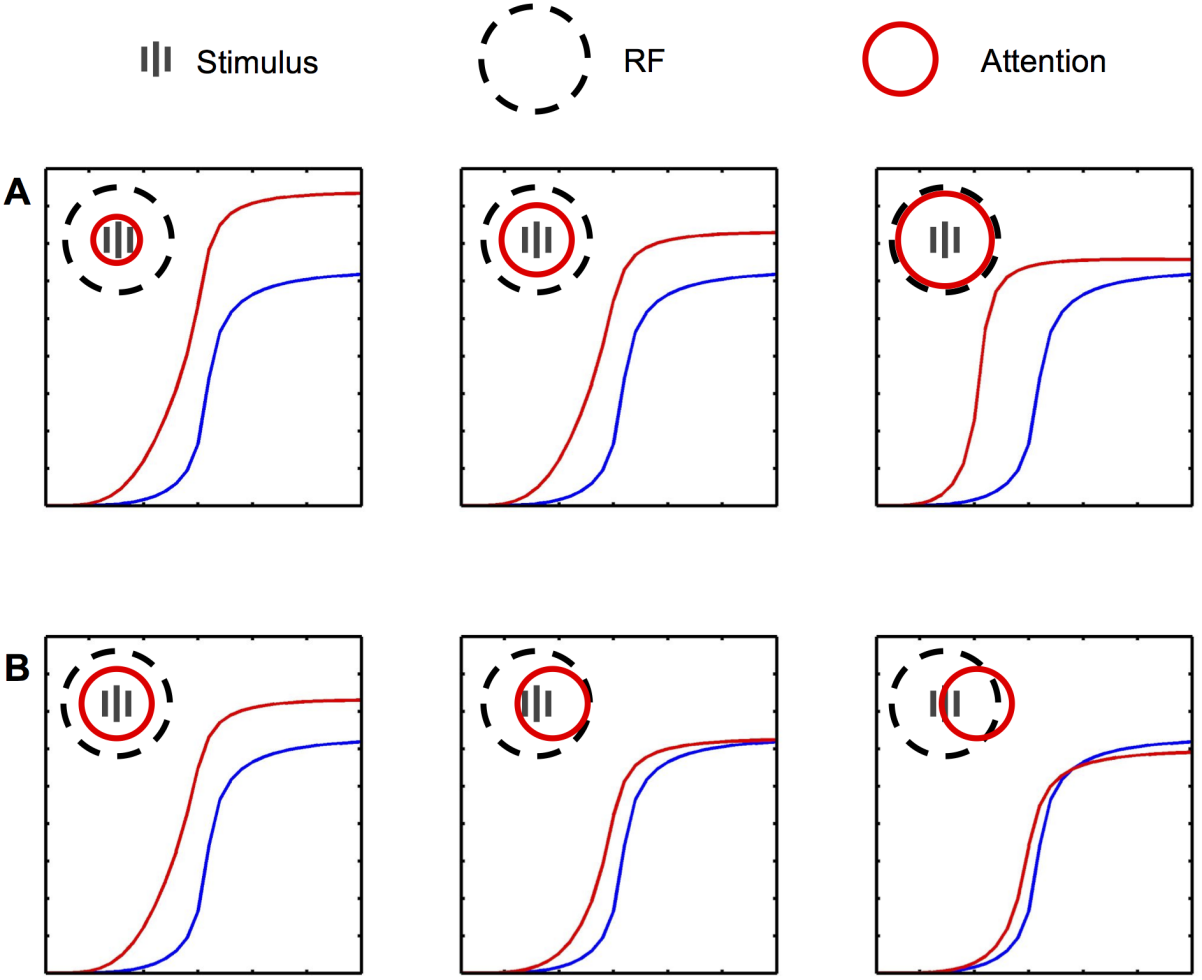
Effect of attention on response curves as a function of stimulus intensity. Top row (A): Similarly to Heeger & Reynolds’ normalization model of attention, response curves become more similar to a response gain or a contrast gain (respectively) when the attentional field is made smaller or larger in comparison to RF size. Middle row (B): In addition, the model predicts that attentional effects should shift from a response gain (first panel) to a contrast gain (second panel), and might also produce crossing curves (third panel), as the focus of attention shifts away from the center of the stimulus and of the cell’s RF. Note that the contrast-gain effect persists (though with decreasing magnitude) as attention shifts further away from the RF, due to the wide range of feedback connections between top and input layers. Compare with Fig 5 of [7].

We also studied the effects of attentional modulation on contrast response curves, that is, the curve of neural response as a function of stimulus contrast (Fig 6). The stimulus is again a single semi-rectified Gabor function centered at the center of the cell’s RF, with stimulus amplitude increasing linearly until neural response saturates. We confirmed that our model exhibits the phenomenon predicted by Reynolds and Heeger’s normalization model, in which the modulation of response curves resembles more a response-gain or an input-gain, as the spatial size of the attentional modulation is made smaller or larger (respectively) in regard to RF size (Fig 6A, top row). Intuitively this is because larger attentional modulation implies that neighbouring cells are also modulated with almost equal strength; thus the net effect of attention, after taking into account inhibitory normalization between neighbouring cells, is very small at high contrasts. However at lower contrasts, normalization is inherently less potent: the denominator of the normalization equation (Eqs 3 and 6) is dominated by the additive constant and thus produces relatively little mutual inhibition, leaving the attentional multiplication relatively untouched.

In addition, our model makes the novel prediction that the modulation of response curves should move gradually from a response gain to an input gain, as the focus of attention moves away from the center of the cell’s RF (Fig 6B, bottom row). Intuitively, this effect can be explained as follows: as the focus of attention moves away from the cell’s RF center, the cell feels less effect from the small-radius top-down multiplicative modulation (which produces the response gain); however, it still receives the effects of indirectly-modulated bottom-layer inputs (which produce a contrast / input-gain), because the latter effect has a much larger spatial radius, due to the lateral range of feedback and feedforward connections. A curious observation is that, for a certain distance between attentional focus and RF center, the model produces intersecting curves, as was reported in some studies (Fig 6c; see also [7]). In our model, this is again a direct consequence of the dependence of normalization on contrast: as contrast increases, the relatively small increase from modulated inputs is defeated by the increased inhibition from neighbours which now receive stronger, direct top-down modulation than the studied cell.

### Attentional effects on receptive fields

**Fig 7:**
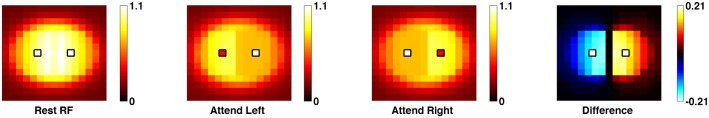
Spatial attention shifts receptive fields. Reconstructed receptive fields obtained by mapping the response of the cell to a moving probe stimulus, under various attentional conditions. Compare with Fig 2 of [13].

**Fig 8:**
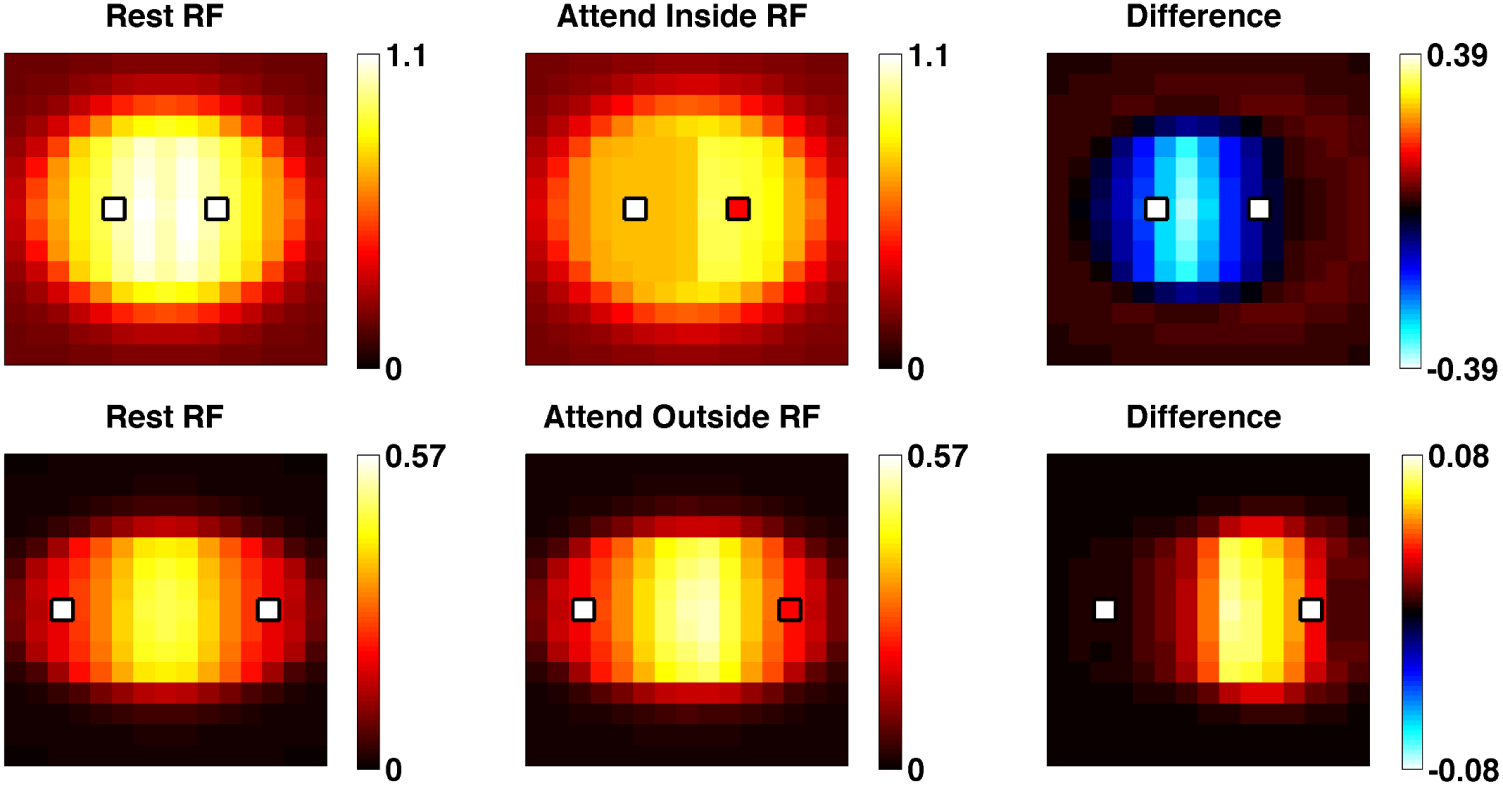
Spatial attention resizes receptive fields. Conventions are as in Fig 7. Focusing attention inside the receptive field shifts and shrinks the receptive field around the focus of attention (top row). Focusing attention just outside the receptive field expands it slightly towards the focus of attention (bottom row). Compare with Fig 6 of [15].

We investigated the effects of attention on RF position (Fig 7) and size (Fig 8). Our simulated setting is similar to the one described by [13,15]. Two “target” stimuli (Gabor patches) are positioned on both sides of the studied cell’s location. Following [13,15], these are of the anti-preferred orientation and at reduced intensity (85%) to avoid saturating the cell’s response. Then, a “probe” stimulus (preferred orientation, full intensity) is positioned successively at 15x15 locations arranged in a square grid centered at the cell’s location. The responses of the cell to each of these probe stimuli are collated to build a map of the cell’s actual RF in a given condition.

We first position the “target” stimuli well within the studied cell’s rest RF, and compute the effective RF when attention is focused on either of the targets, as well as in the absence of attention. Results in Fig 7 show that the cell’s rest RF is both shifted in position and shrunk in size by focusing attention on a position within the RF. The RF shift is also illustrated by comparing conditions in which attention is focused on either of the two targets. By contrast, when the two targets are located on the outer edge of the RF, focusing attention on a target expands the RF towards it rather than shrinking it, reproducing the results of [15].

Finally, David and colleagues showed that feature-based attention can also subtly alter the shape preference of V4 cells (that is, their featural receptive field), making them closer to the attended features in the spectral domain [17]. We use a procedure similar to theirs to reconstruct the so-called “spectral receptive field” (SRF) of top-layer cells, which provides a visualization of featural receptive fields. The SRF is essentially a normalized two-dimensional Fourier transform of the cell’s preferred stimuli, indicating the response as a function of stimulus vertical and horizontal frequency (or, equivalently, orientation and overall spatial frequency). It is computed by reverse correlation, as a weighted average of the 2D Fourier transforms of incoming stimuli, weighted by the responses to this stimuli (David and colleagues include a correction term for spectral autocorrelation, which we neglect here for simplicity).

**Fig 9:**
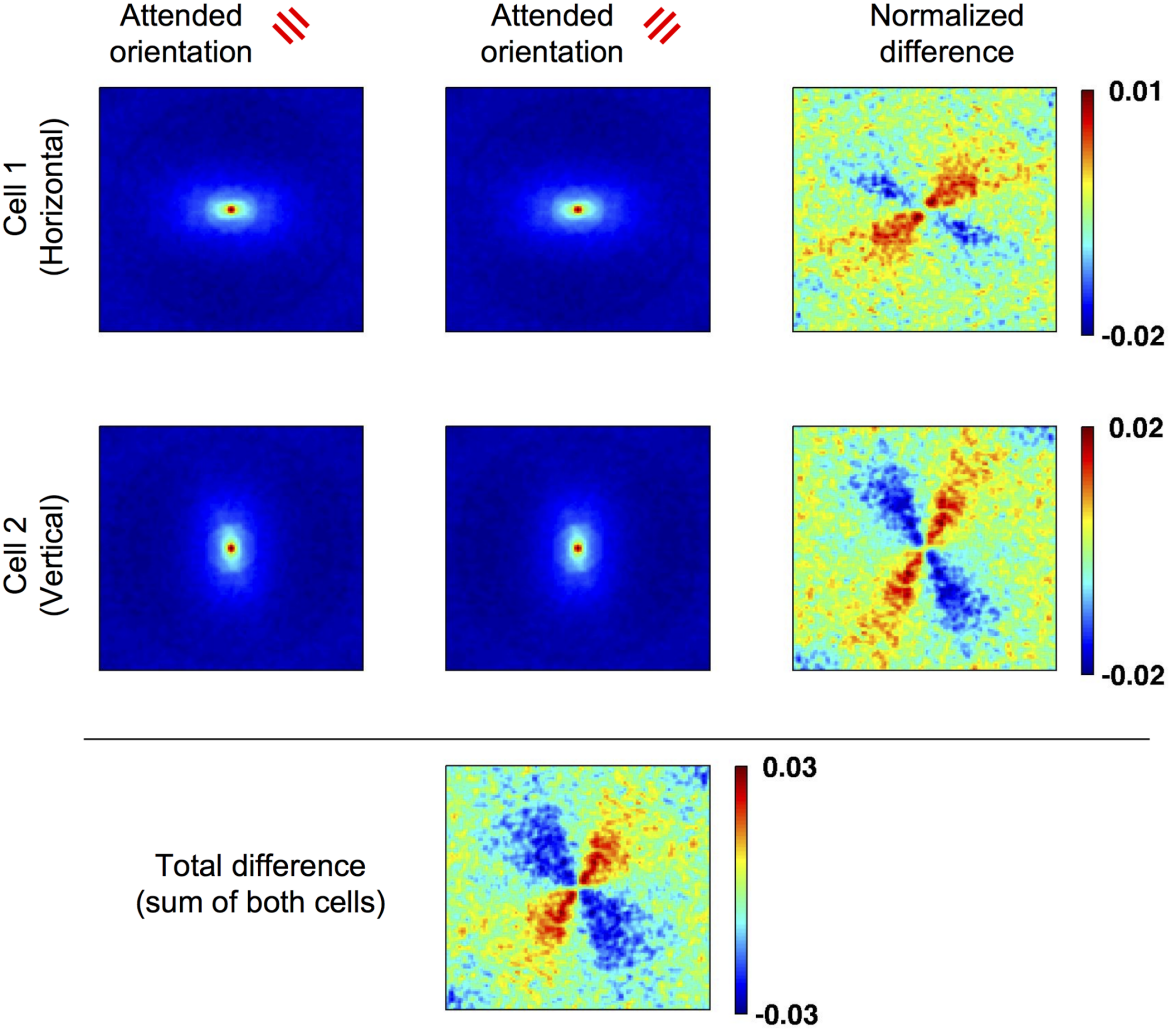
Feature-based attention shifts the reconstructed receptive fields of top-layer cells in featural (spectral) space. Top and Middle row: the reconstructed spectral preference (2D Fourier spectrum) of a horizontally-selective and vertically-selective cell, respectively, under attention to two different orientations. Note that both cells have preferred orientation equidistant from either attended orientation, and thus receive quantitatively similar attentional modulation in all conditions. The last column indicates the normalized difference (a-b) / (a+b) between the two attentional conditions, reflecting the shift caused by attention. Bottom panel: difference between attentional conditions, using the summed spectral receptive fields of both horizontally- and vertically-selective cells.

We computed the SRF of top-layer cells by exposing them to a number of image patches, taken from the Van Hateren database of whitened images (thought to emulate the output of retinal processing), and then weighting the summed 2D Fourier transforms of these patches by the cell’s response (Fig 9). The resulting SRFs are as expected from the known feature preference of the cell, consisting of elongated ellipses aligned to the cell’s preferred orientation. Crucially, applying attention to a particular orientation has the effect of subtly shifting the RFs towards this orientation, as seen from the difference maps. This reproduces the effects observed by David and colleagues [17].

## Discussion

We have shown that many effects of attention on neural responses can be explained by a relatively simple mechanism, invoking modulatory feedback connections between areas and short-range lateral inhibition within each area. Our model replicates observed attentional effects on both firing rates and receptive field structure, including both response and contrast gain effects, biased competition scaled to RF size, feature-similarity gain modulation, RF shifts and rescaling according to position of attentional focus, and shifts of featural tuning.

Our model makes the novel prediction that attentional effects on contrast response curves should change from response gain to contrast gain as the focus of attention shifts away from the cell’s RF center. We are not aware of any reported study that would provide a direct test of this prediction. However, we note that Herman and colleagues [38] estimated the effect of attention focused either on a fixed-position target, or to smaller targets with variable, unpredictable position. They reported that behavioral responses were consistent with a response-gain effect when the attended stimulus position was known and predictable, and a contrast-gain effect when this position was unknown and unpredictable (within a fixed region). They interpret this result as an effect of a larger attentional field size, supported by imaging data. However we note that this result is also consistent with a larger distance between the attentional focus and the exact stimulus position (due to increased uncertainty in stimulus position). This could also produce a larger area of increased activity in imaging data if we assume that the attentional focus moved over possible stimulus positions before stimulus onset. Further experimental work is clearly needed to provide a clear test of this prediction.

While our model makes use of “feedback” connections, we use the term in a functional rather than anatomic sense: by feedback connections, we mean any topographically organized connections between “higher” and “lower” areas in the visual hierarchy. While direct cortico-cortical connections may well support much of this feedback influence, sub-cortical structures also carry targeted, topographically precise “feedback” information between cortical areas. For example, the pulvinar thalamic nucleus contains multiple maps that receive overlapping projections from hierarchically adjacent cortical areas, and has been implicated in attentional processing [29,39]. Thus, both cortico-cortical and cortico-thalamo-cortical projections may support the feedback projections discussed here.

Our model implements simple neurons, with instantaneous activation values, rather than individual spikes or time-varying firing rates. Therefore, although it can reproduce gross temporal dynamics at the scale of whole areas, it cannot capture the fine temporal dynamics of attentional effects on individual cell responses. In particular, it cannot describe known effects of attention on firing synchrony [40] and noise decorrelation [41], or the stronger effect of attention on the late phase of neural responses. Although the model captures diverse effects on firing rates and receptive field structure, investigating fine temporal and correlation effects will require more advanced models with realistic temporal dynamics.

Because our model is based on feedback connections and mutual inhibition within each layer, it is clearly related to Tsotsos’ selective tuning model [42]. However, our model aims at a more biologically plausible implementation that does not invoke specific operations such as connection pruning or biased winner-take-all. Furthermore, the source of attentional modulation in our model is a plain response modulation of the “top” layer (which models the intermediate layers of the visual cortex, namely V4 and MT); this is in contrast with most existing models of attentional effects, which tend to posit that attention operates through gain modulation of inputs to the studied layer [4,5,19,21]. As mentioned in the introduction, substantial evidence suggests that attentional modulation falls on higher rather than input layers of the visual system, including a top-down cascade of attentional effects [24], decreasing size of attentional modulation in upstream areas [9], direct interactions between attentional and mid-level visual areas during attention [31,33], and strong connections from FEF to V4 rather than V1 or V2 [30].

The model discusses within-RF competitive effects, which are known to be scaled to RF size across many areas [9,26]. We show that this apparently fine-tuned matching emerges automatically from short-range mutual inhibition within each layer. However, our model does not include the distinct phenomenon of outside-RF surround suppression. Surround suppression differs from within-RF competition in many ways, perhaps more spectacularly by being maximal for iso-oriented stimuli rather than anti-oriented stimuli. The mechanisms for within-RF and surround suppression are likely to differ [43], and the mechanism for surround suppression remains largely unclear (see the apparently conflicting results of [44] and [45]; also [46] and [47]). More importantly for our purposes, attentional effects for surround stimuli are known to be weak in comparison to the strong and reliable within-RF competitive effect [9]. Therefore we chose to omit surround suppression from our model at the present time and postpone integration of surround effects for future work.

An important aspect of our model is the paucity of assumptions, especially when considering the wide range of effects explained. We only posit the existence of modulatory feedback connections and short-range mutual inhibition between neighbouring neurons; these two effects suffice to produce diverse, yet precise effects, including complex alterations of response rates and receptive fields, automatically scaled to RF size across varying areas. In particular, there is no need for a dedicated circuitry that would selectively target spatial and fine-feature-specific neuronal populations in early visual cortex. Importantly, this does not exclude the possibility that such specific mechanisms are also involved in generating attentional modulation. Rather, it suggests that these mechanisms need not be invoked to explain the set of effects described above. In this respect, our model may thus be seen as a potential “null model” of attentional modulation in cortical responses, describing observed attentional effects as the expected outcome of well-known features of cortical organization.

The model supposes that a top-down attentional signal falls on the higher levels of the visual system, in the form of a multiplicative modulation; however, it is agnostic as to the source of this top-down signal, or how it is computed. A large body of research suggests that allocation of visual attention is controlled by a set of mutually connected areas, including the Frontal Eye Fields, Lateral Intraparietal cortex, and sub-cortical structures such as the Superior Colliculus [1,2,29] or the pulvinar [39]. Several models seek to explain how these areas compute the actual locus of attention under different conditions, including bottom-up saliency effects [48] and guided target-seeking search [49]. Integrating such models of attentional selection with reliable mechanistic models of attentional modulation could open the way to a broad understanding of visual attention in the brain.

## Materials and Methods

Here we provide a full description of our model. We provide both descriptive equations, and a snippet of our original Matlab code for reference. The full Matlab source code for our implementation of the model is available online.

Our model is composed of two reciprocally-connected square areas (the “top” and “bottom” layers). The bottom layer emulates the properties of visual area V1, while the top layer emulates properties of higher visual areas that receive the top-down attentional modulation (such as V4 and MT).

Each area is organized as a regular lattice of locations, with one location above every pixel of the input image, and contain 8 orientation-selective cells at every location. In the bottom layer, the cells at any location are a full complement of orientation-selective edge detectors, implemented as Gabor filters of 8 orientations between 0 and 157.5 degrees inclusive. Thus, the total *excitation* of a bottom-layer cell at position x and orientation θ is:

$$E_{x,\theta }^{Bottom}={\left(\;\left(1\;+\;FB_{x,\theta }\right)\;*\;Gabor\left(x,\;\theta ,\;\sigma^{Bottom}\right)⊗Image\right)}^{2}$$ (1)

where *Gabor*(x, θ, *σ*^*Bottom*^) represents a Gabor filter centered at position x with orientation θ and standard deviation (“radius”) *σ*^*Bottom*^, and ⊗ represents spatial convolution (i.e. pointwise multiplication followed by summation). Note that *σ*^*Bottom*^, which determines the effective size of V1 RFs, was always set to 3 pixels in all experiments. The 1+FB term represents modulatory feedback from the top layer, as described below (the additive constant 1 ensures that feedback is strictly excitatory). Note that cell activations are squared to reproduce a power-law response curve [50,51].

The model implements divisive, non-feature-specific mutual inhibition (i.e. normalization) between neighbouring cells within each area, again with a Gaussian profile. The normalized excitation defines the cell’s final response $R_{x,\theta }^{Bottom}$:

$$Inh^{Bottom}\left(x\right)\;=\;\Sigma_{\theta }(E_{x,\theta }^{Bottom}⊗Gauss\left(x,\;\sigma^{Inh}\right))$$ (2)

$$R_{x,\theta }^{Bottom}=\frac{E_{x,\theta }^{Bottom}}{Inh^{Bottom}\left(x\right)\;+\;C^{Bottom}}$$ (3)

*Gauss(x*,*σ)* denotes a Gaussian function with mean x and standard deviation σ. Note that Eq 2 essentially denotes a convolution with a Gaussian kernel for each orientation, then a pointwise summation of the resulting maps (reflecting short-range, untuned mutual inhibition). Eq 3, in which *c*^*Bottom*^ is a constant, is the well-known normalization equation [4,43,52]. In all simulations, *σ*^*Inh*^ was set to 1, emphasizing the short range of inhibition in our model.

Top-layer cells receive excitatory connections from the bottom-layer cells: the $R_{x,\theta }^{Bottom}$ values constitute the inputs to the top layer. The connection between any two cells is proportional to a Gaussian function of their distance, both in space and in preferred orientation. Note that while bottom-layer cells are orientation-selective due to the Gabor filters that constitute their input weights, the orientation selectivity of top-layer cells arises solely from their selective connections from bottom-layer cells. Furthermore, top-layer cells also receive the attentional modulation, which is either spatial or feature-based (i.e. orientation-specific). Normalization occurs as in the bottom layer.

$$E_{x,\theta }^{Top}={\left(\;\left(1\;+\;A_{x,\theta }\right)\;*\;Gauss\left(\theta ,\;\sigma^{Ori}\right)⊗\;Gauss\left(x,\;\sigma^{Top}\right)⊗\;R_{x,\theta }^{Bottom}\right)}^{2}$$ (4)

$$Inh^{Top}\left(x\right)\;=\;\Sigma_{\theta }(E_{x,\theta }^{Top}⊗Gauss\left(x,\;\sigma^{Inh}\right))$$ (5)

$$R_{x,\theta }^{Top}=\frac{E_{x,\theta }^{Top}}{Inh^{Top}\left(x\right)\;+\;C^{Top}}$$ (6)

Notice that the first of these equations describes a convolution of the bottom-layer maps with both spatial and orientation Gaussian kernels, with pointwise multiplication by the local attentional field *A*_*x*,*θ*_ (see below). In other words, the Bottom-layer output is convolved with a Gaussian spatial kernel, and the resulting set of maps (one per orientation) is then convolved with an orientation-wise 1D Gaussian kernel centered at the appropriate orientation for each given top-layer cell (and with standard deviation *σ*^*Ori*^). Again, activations are squared, and attentional feedback is made purely excitatory by a unit additive constant. The other two equations describe local normalization, similarly to Eq 3 above. Note that *σ*^*Inh*^ has the same value (i.e. 1) for both layers.

The attentional term *A*_*x*,*θ*_ is a Gaussian function of either the distance from attentional focus *X*_*att*_ (for spatial attention) or the angular distance between the preferred orientation and the attended orientation (for feature-based attention).

$$A_{x}=\alpha_{Spatial}^{Att}Gauss(X_{Att},\;\sigma^{AttSpat})$$ (7)

$$A_{\theta }=\alpha_{Feature}^{Att}Gauss\left(\theta_{Att},\sigma^{Ori}\right)$$ (8)

Notice that the spread of feature-based attention in orientation space (Eq 8) is equal to *σ*^*Ori*^, the spread of connection selectivity in orientation space, as used in Eqs 4 and 9.

In addition, top-layer cells send feedback connections to bottom-layer cells, as represented by the *FB* term in Eq 3. The feedback connection between any two cells has weight proportional to the feedforward connection between these two cells. Thus, the expression that computes the *FB*_*x*,*θ*_ term in Eq 1 is similar to Eq 4, replacing *R*^*Bottom*^ with *R*^*Top*^ and multiplying by a constant α^FB^ indicating the relative “strength” of feedback:

$$FB_{x,\theta }^{Top}=\alpha^{FB}\;*\;Gauss\left(\theta ,\;\sigma^{Ori}\right)⊗\;Gauss\left(x,\;\sigma^{Top}\right)⊗\;R_{x,\theta }^{Top}$$ (9)

As a result of top-down feedback connections, bottom-layer responses are modified, so the activities of top-layer cells (which use bottom-layer cells as inputs) must be recomputed. The whole process is iterated until equilibrium is reached (in our experiments, 30 iterations suffice to equilibrate responses).

Note that we used the exact same values for all model parameters across all of our experiments. These were set to *σ*^*Bottom*^ = 3, *σ*^*Top*^ = 12, *σ*^*Inh*^ = 1, *σ*^*AttSpat*^ = 3, *σ*^*Ori*^ = 1.1*π/8, $\alpha_{Spatial}^{Att}$ = 2, $\alpha_{Feature}^{Att}$ = 0.2, α^FB^ = 18.

To determine adequate values for the normalization constants *c*^*Bottom*^ and *c*^*Top*^, we ran the model on a specific input image composed of a semi-rectified Gabor (identical to the ones used for the biased competition experiments described in Results), setting the values of *c* for each layer to a fixed multiple *σ*^*Norm*^ of the maximum pre-inhibition activation in that layer at each successive iteration. This fixed multiplier *σ*^*Norm*^ = 0.6 therefore indirectly determines both *c*^*Bottom*^ and *c*^*Top*^. The values of *c*^*Bottom*^ and *c*^*Top*^ quickly converged to 2.6757 and 1.09*10^−4^, respectively. These values were used for all experiments.

Note that top-layer RFs are 4 times as large as bottom-layer RFs, reflecting the rough relative size of V1 and V4/MT RFs; while inhibition has a (constant) range much shorter than either RF size or attentional modulation, consistent with the short, layer-independent range of inhibition posited by our model.

To estimate the sensitivity of our model to precise parameter values, we simply altered all free parameters (*σ*^*Bottom*^,*σ*^*Top*^,*σ*^*Inh*^,*σ*^*AttSpat*^,*σ*^*Ori*^,$\alpha_{Spatial}^{Att}$,$\;\alpha_{Feature}^{Att}$,α^FB^,*σ*^*Norm*^) by a common multiple. We find that cutting all values by 20% does not modify the qualitative conclusions of the model (S1 Fig). However, increasing all parameter values by 20% (but not 10%) effectively eliminates the attentional biasing effect. Thus, the model is moderately tolerant to parameter changes, despite the numerous nonlinear interactions present in the model. Again, we stress that we used the same parameter values for all experiments, in contrast to other models in which parameters are adapted to produce adequate results for each experiment; we believe this strengthens the plausibility of our model.

Due to the simplicity of the model, we can complement the above descriptive equations with a snippet of our actual Matlab code, thus providing an unambiguous description of the model’s operation (note that our full code is available online).

% Network activities are computed iteratively to allow

% feedback to equilibrate

for numiter = 1:30

 % excFFv1 is the network input (dimensions ImWidth x ImHeight x 8),

 % computed by processing the stimulus through a bank of Gabor

 % filters at 8 different orientations

 v1 = excFFV1; % Stimulus input

 % Feedback from top layer (initially, fbv1 = 0)

 v1 = v1.* (1 + fbv1);

 v1 = v1.^ 2; % Squaring

 % Normalization (division by local activity summed over orientations)

 v1inh = imfilter (sum(v1,3), INHF); % INHF is a 2D Gaussian filter

 v1 = bsxfun(@rdivide, v1, (SIGMAV1 + v1inh));

 % Excitatory transmission from bottom to top layer.

 % Note the use of separable convolution for Gaussian filters.

 % RFV4_1D is a 1-dimensional Gaussian filter, applied in the x and y

 % dimension. ORIF is a 1-dimensional Gaussian filter applied in the

 % third (orientation) dimension.

 v4 = imfilter(imfilter(v1, RFV4_1D), RFV4_1D');

 v4 = imfilter(v4, ORIF, 'circular');

 %Attentional modulation

 v4 = bsxfun(@times, v4, (1 + v4attfield));

 v4 = v4.^ 2; % Squaring

 % Normalization by local activity

 v4inh = imfilter (sum(v4,3), INHF);

 v4 = bsxfun(@rdivide, v4, (SIGMAV4 + v4inh));

 % Computation of the feedback to the bottom layer for next step

 fbv1 = imfilter(imfilter(v4, RFV4_1D), RFV4_1D');

 fbv1 = FBSTRENGTH * imfilter(fbv1, ORIF, 'circular');

end

## Supporting Information

S1 Fig Reducing all free parameters by 20% preserves the conclusions of the model. Conventions are as in Figs 3, 5, 7 and 8. While the quantitative values differ, the effects of attention (biased competition, feature-similarity gain, RF shifts and RF scalings) are qualitatively preserved. Note that, by contrast, increasing all parameter values by 20% (but not 10%) largely eliminates most of the attentional effects.
(TIFF)

## References

[1] Carrasco M. Visual attention: The past 25 years. Vision Res. 2011;51: 1484–1525. 10.1016/j.visres.2011.04.012 21549742 PMC3390154

[2] Noudoost B, Chang MH, Steinmetz NA, Moore T. Top-down control of visual attention. Curr Opin Neurobiol. 2010;20: 183–190. 10.1016/j.conb.2010.02.003 20303256 PMC2901796

[3] Reynolds JH, Chelazzi L. Attentional modulation of visual processing. Annu Rev Neurosci. 2004;27: 611–647. 15217345 10.1146/annurev.neuro.26.041002.131039

[4] Reynolds JH, Heeger DJ. The Normalization Model of Attention. Neuron. 2009;61: 168–185. 10.1016/j.neuron.2009.01.002 19186161 PMC2752446

[5] Reynolds JH, Pasternak T, Desimone R. Attention Increases Sensitivity of V4 Neurons. Neuron. 2000;26: 703–714. 10896165 10.1016/s0896-6273(00)81206-4

[6] Martínez-Trujillo J, Treue S. Attentional modulation strength in cortical area MT depends on stimulus contrast. Neuron. 2002;35: 365–370. 12160753 10.1016/s0896-6273(02)00778-x

[7] Williford T, Maunsell JHR. Effects of spatial attention on contrast response functions in macaque area V4. J Neurophysiol. 2006;96: 40–54. 16772516 10.1152/jn.01207.2005

[8] Desimone R. Visual attention mediated by biased competition in extrastriate visual cortex. Philos Trans R Soc Lond B Biol Sci. 1998;353: 1245–1255. 9770219 10.1098/rstb.1998.0280 PMC1692333

[9] Luck SJ, Chelazzi L, Hillyard SA, Desimone R. Neural Mechanisms of Spatial Selective Attention in Areas V1, V2, and V4 of Macaque Visual Cortex. J Neurophysiol. 1997;77: 24–42. 9120566 10.1152/jn.1997.77.1.24

[10] Kastner S, Ungerleider LG. The neural basis of biased competition in human visual cortex. Neuropsychologia. 2001;39: 1263–1276. 11566310 10.1016/s0028-3932(01)00116-6

[11] Kastner S, Weerd PD, Desimone R, Ungerleider LG. Mechanisms of Directed Attention in the Human Extrastriate Cortex as Revealed by Functional MRI. Science. 1998;282: 108–111. 9756472 10.1126/science.282.5386.108

[12] Martinez-Trujillo JC, Treue S. Feature-Based Attention Increases the Selectivity of Population Responses in Primate Visual Cortex. Curr Biol. 2004;14: 744–751. 15120065 10.1016/j.cub.2004.04.028

[13] Womelsdorf T, Anton-Erxleben K, Pieper F, Treue S. Dynamic shifts of visual receptive fields in cortical area MT by spatial attention. Nat Neurosci. 2006;9: 1156–1160. 16906153 10.1038/nn1748

[14] Connor CE, Preddie DC, Gallant JL, Essen DCV. Spatial Attention Effects in Macaque Area V4. J Neurosci. 1997;17: 3201–3214. 9096154 10.1523/JNEUROSCI.17-09-03201.1997 PMC6573654

[15] Anton-Erxleben K, Stephan VM, Treue S. Attention Reshapes Center-Surround Receptive Field Structure in Macaque Cortical Area MT. Cereb Cortex. 2009;19: 2466–2478. 10.1093/cercor/bhp002 19211660 PMC2742598

[16] Motter BC. Neural correlates of attentive selection for color or luminance in extrastriate area V4. J Neurosci. 1994;14: 2178–2189. 8158264 10.1523/JNEUROSCI.14-04-02178.1994 PMC6577115

[17] David SV, Hayden BY, Mazer JA, Gallant JL. Attention to Stimulus Features Shifts Spectral Tuning of V4 Neurons during Natural Vision. Neuron. 2008;59: 509–521. 10.1016/j.neuron.2008.07.001 18701075 PMC2948549

[18] Hamker FH. The Reentry Hypothesis: The Putative Interaction of the Frontal Eye Field, Ventrolateral Prefrontal Cortex, and Areas V4, IT for Attention and Eye Movement. Cereb Cortex. 2005;15: 431–447. 15749987 10.1093/cercor/bhh146

[19] Hamker FH, Zirnsak M. V4 receptive field dynamics as predicted by a systems-level model of visual attention using feedback from the frontal eye field. Neural Netw. 2006;19: 1371–1382. 17014990 10.1016/j.neunet.2006.08.006

[20] Reynolds JH, Chelazzi L, Desimone R. Competitive mechanisms subserve attention in macaque areas V2 and V4. J Neurosci. 1999;19: 1736–1753. 10024360 10.1523/JNEUROSCI.19-05-01736.1999 PMC6782185

[21] Womelsdorf T, Anton-Erxleben K, Treue S. Receptive Field Shift and Shrinkage in Macaque Middle Temporal Area through Attentional Gain Modulation. J Neurosci. 2008;28: 8934–8944. 10.1523/JNEUROSCI.4030-07.2008 18768687 PMC6670861

[22] Ghose GM. Attentional modulation of visual responses by flexible input gain. J Neurophysiol. 2009;101: 2089–2106. 10.1152/jn.90654.2008 19193776 PMC2695627

[23] Lee J, Maunsell JHR. A normalization model of attentional modulation of single unit responses. PLoS One. 2009;4: e4651 10.1371/journal.pone.0004651 19247494 PMC2645695

[24] Buffalo EA, Fries P, Landman R, Liang H, Desimone R. A backward progression of attentional effects in the ventral stream. Proceedings of the National Academy of Sciences. 2010;107: 361–365. 10.1073/pnas.0907658106 PMC2806732 20007766

[25] Maunsell JHR, Cook EP. The role of attention in visual processing. Philos Trans R Soc Lond B Biol Sci. 2002;357: 1063–1072. 12217174 10.1098/rstb.2002.1107 PMC1693016

[26] Kastner S, De Weerd P, Pinsk MA, Elizondo MI, Desimone R, Ungerleider LG. Modulation of sensory suppression: implications for receptive field sizes in the human visual cortex. J Neurophysiol. 2001;86: 1398–1411. 11535686 10.1152/jn.2001.86.3.1398

[27] Roelfsema PR, Lamme VA, Spekreijse H. Object-based attention in the primary visual cortex of the macaque monkey. Nature. 1998;395: 376–381. 9759726 10.1038/26475

[28] Thiele A, Pooresmaeili A, Delicato LS, Herrero JL, Roelfsema PR. Additive effects of attention and stimulus contrast in primary visual cortex. Cereb Cortex. 2009;19: 2970–2981. 10.1093/cercor/bhp070 19372142 PMC2774399

[29] Baluch F, Itti L. Mechanisms of top-down attention. Trends Neurosci. 2011;34: 210–224. 10.1016/j.tins.2011.02.003 21439656

[30] Barone P, Batardiere A, Knoblauch K, Kennedy H. Laminar distribution of neurons in extrastriate areas projecting to visual areas V1 and V4 correlates with the hierarchical rank and indicates the operation of a distance rule. J Neurosci. 2000;20: 3263–3281. 10777791 10.1523/JNEUROSCI.20-09-03263.2000 PMC6773101

[31] Gregoriou GG, Gotts SJ, Zhou H, Desimone R. High-frequency, long-range coupling between prefrontal and visual cortex during attention. Science. 2009;324: 1207–1210. 10.1126/science.1171402 19478185 PMC2849291

[32] Moore T, Armstrong KM. Selective gating of visual signals by microstimulation of frontal cortex. Nature. 2003;421: 370–373. 12540901 10.1038/nature01341

[33] Saalmann YB, Pigarev IN, Vidyasagar TR. Neural Mechanisms of Visual Attention: How Top-Down Feedback Highlights Relevant Locations. Science. 2007;316: 1612–1615. 17569863 10.1126/science.1139140

[34] Lauritzen TZ, D’Esposito M, Heeger DJ, Silver MA. Top–down flow of visual spatial attention signals from parietal to occipital cortex. J Vis. 2009;9: 18. 10.1167/9.13.18 PMC2857595 20055551

[35] Deco G, Rolls ET. A neurodynamical cortical model of visual attention and invariant object recognition. Vision Res. 2004;44: 621–642. 14693189 10.1016/j.visres.2003.09.037

[36] Montijn JS, Klink PC, van Wezel RJA. Divisive normalization and neuronal oscillations in a single hierarchical framework of selective visual attention. Front Neural Circuits. 2012;6: 22 10.3389/fncir.2012.00022 22586372 PMC3343306

[37] Compte A, Wang X-J. Tuning curve shift by attention modulation in cortical neurons: a computational study of its mechanisms. Cereb Cortex. 2006;16: 761–778. 16135783 10.1093/cercor/bhj021

[38] Herrmann K, Montaser-Kouhsari L, Carrasco M, Heeger DJ. When size matters: attention affects performance by contrast or response gain. Nat Neurosci. 2010;13: 1554–1559. 10.1038/nn.2669 21057509 PMC3058765

[39] Saalmann YB, Pinsk MA, Wang L, Li X, Kastner S. The Pulvinar Regulates Information Transmission Between Cortical Areas Based on Attention Demands. Science. 2012;337: 753–756. 10.1126/science.1223082 22879517 PMC3714098

[40] Bosman CA, Schoffelen J-M, Brunet N, Oostenveld R, Bastos AM, Womelsdorf T, et al Attentional Stimulus Selection through Selective Synchronization between Monkey Visual Areas. Neuron. 2012;75: 875–888. 10.1016/j.neuron.2012.06.037 22958827 PMC3457649

[41] Cohen MR, Maunsell JHR. Attention improves performance primarily by reducing interneuronal correlations. Nat Neurosci. 2009;12: 1594–1600. 10.1038/nn.2439 19915566 PMC2820564

[42] Tsotsos JK, Culhane SM, Kei Wai WY, Lai Y, Davis N, Nuflo F. Modeling visual attention via selective tuning. Artif Intell. 1995;78: 507–545.

[43] Carandini M, Heeger DJ. Normalization as a canonical neural computation. Nat Rev Neurosci. 2011; 10.1038/nrn3136 PMC3273486 22108672

[44] Haider B, Krause MR, Duque A, Yu Y, Touryan J, Mazer JA, et al Synaptic and network mechanisms of sparse and reliable visual cortical activity during nonclassical receptive field stimulation. Neuron. 2010;65: 107–121. 10.1016/j.neuron.2009.12.005 20152117 PMC3110675

[45] Ozeki H, Finn IM, Schaffer ES, Miller KD, Ferster D. Inhibitory stabilization of the cortical network underlies visual surround suppression. Neuron. 2009;62: 578–592. 10.1016/j.neuron.2009.03.028 19477158 PMC2691725

[46] Adesnik H, Bruns W, Taniguchi H, Huang ZJ, Scanziani M. A neural circuit for spatial summation in visual cortex. Nature. 2012;490: 226–231. 10.1038/nature11526 23060193 PMC3621107

[47] Self MW, Lorteije JAM, Vangeneugden J, van Beest EH, Grigore ME, Levelt CN, et al Orientation-tuned surround suppression in mouse visual cortex. J Neurosci. 2014;34: 9290–9304. 10.1523/JNEUROSCI.5051-13.2014 25009262 PMC6608354

[48] Itti L, Koch C. Computational modelling of visual attention. Nat Rev Neurosci. 2001;2: 194–203. 11256080 10.1038/35058500

[49] Miconi T, Groomes L, Kreiman G. There’s Waldo! A Normalization Model of Visual Search Predicts Single-Trial Human Fixations in an Object Search Task. Cereb Cortex, published online 6 2015. 10.1093/cercor/bhv129 PMC4898665 26092221

[50] Miller KD, Troyer TW. Neural Noise Can Explain Expansive, Power-Law Nonlinearities in Neural Response Functions. J Neurophysiol. 2002;87: 653–659. 11826034 10.1152/jn.00425.2001

[51] Heeger DJ. Modeling simple-cell direction selectivity with normalized, half-squared, linear operators. J Neurophysiol. 1993;70: 1885–1898. 8294961 10.1152/jn.1993.70.5.1885

[52] Carandini M, Heeger DJ. Summation and division by neurons in primate visual cortex. Science. 1994;264: 1333–1336. 8191289 10.1126/science.8191289

