# Supplementary figures and images for: A Feedback Model of Attention Explains the Diverse Effects of Attention on Neural Firing Rates and Receptive Field Structure
Source: PLoS Comput Biol. 2016 Feb 18;12(2):e1004770. doi: 10.1371/journal.pcbi.1004770 (PMC4758641; doi:10.1371/journal.pcbi.1004770)

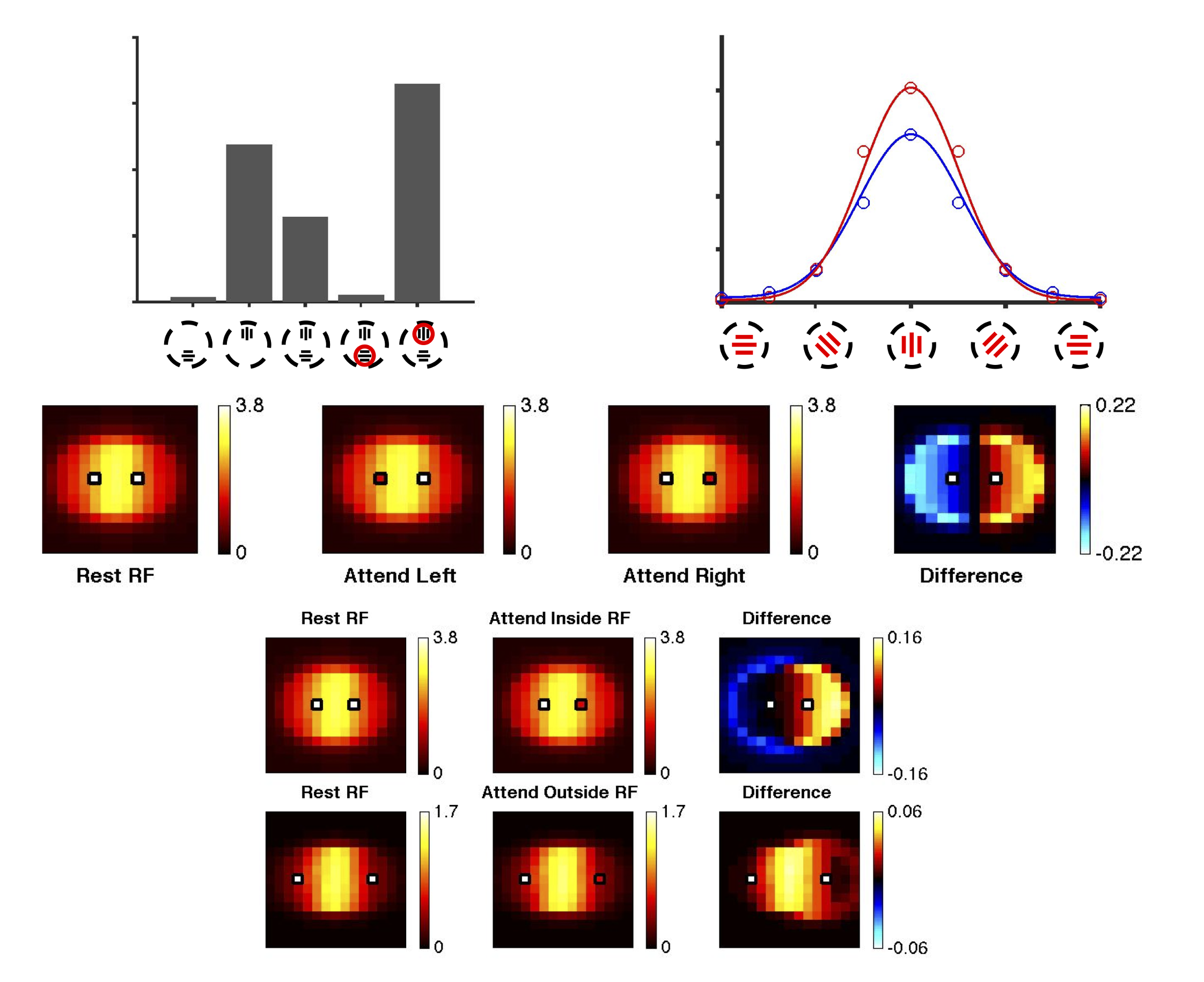

Supplement: S1 Fig — Conventions are as in Figs 3, 5, 7 and 8. While the quantitative values differ, the effects of attention (biased competition, feature-similarity gain, RF shifts and RF scalings) are qualitatively preserved. Note that, by contrast, increasing all parameter values by 20% (but not 10%) largely eliminates most of the attentional effects. (TIFF) [file pcbi.1004770.s001.tiff]
